# Foliar Application of Vegetal-Derived Bioactive Compounds Stimulates the Growth of Beneficial Bacteria and Enhances Microbiome Biodiversity in Lettuce

**DOI:** 10.3389/fpls.2019.00060

**Published:** 2019-02-05

**Authors:** Francesca Luziatelli, Anna Grazia Ficca, Giuseppe Colla, Eva Baldassarre Švecová, Maurizio Ruzzi

**Affiliations:** ^1^Department for Innovation in Biological, Agrofood and Forest Systems, University of Tuscia, Viterbo, Italy; ^2^Department of Agriculture and Forest Sciences, University of Tuscia, Viterbo, Italy

**Keywords:** vegetal protein hydrolysates, *Lactuca sativa* L., plant microbiota, terminal restriction fragment length polymorphism, next generation sequencing, plant growth-promoting bacteria, biocontrol activity

## Abstract

Many studies on plant biostimulants and organic fertilizers have been focused on the ability of these products to increase crop productivity and ameliorate crop tolerance to abiotic stresses. However, little information is available on their effect on plant microbiota, whereas it is well known that microorganisms associated with plant play crucial roles on the health and productivity of their host. The aim of this study was to evaluate the effect of a vegetal-derived protein hydrolysate (PH), a vegetal-derived PH enriched with copper (Cu-PH), and a tropical plant extract enriched with micronutrients (PE) on shoot growth and the epiphytic bacterial population of lettuce plants and the ability of these products to enhance the growth of beneficial or harmful bacteria. The three plant-derived products enhanced shoot biomass of lettuce plants indicating a biostimulant effect of the products. Data obtained using culture-independent (Terminal Restriction Fragment Length Polymorphism and Next Generation Sequencing) and culture-dependent approaches indicated that foliar application of commercial products altered the composition of the microbial population and stimulated the growth of specific bacteria belonging to *Pantoea*, *Pseudomonas*, *Acinetobacter*, and *Bacillus* genus. Data presented in this work demonstrated that some of these strains exhibited potential plant growth-promoting properties and/or biocontrol activity against fungi and bacteria phytopathogens including *Fusarium*, *Trichoderma*, and *Erwinia* species. No indication of potential health risks associated to the enrichment of human or plant bacterial pathogens emerged by the analysis of the microbiota of treated and no-treated plants. Overall, the findings presented in this study indicate that the commercial organic-based products can enhance the growth of beneficial bacteria occurring in the plant microbiota and signals produced by these bacteria can act synergistically with the organic compounds to enhance plant growth and productivity.

## Introduction

Plants provide a rich and diverse habitat which harbors a wide array of bacteria most of which contribute to the growth, and health of their plant hosts (Berendsen et al., 2012; Hirsch and Mauchline, 2012; Jackson et al., 2013; Leff and Fierer, 2013). Most microorganisms in the phyllosphere have ability to promote plant growth through different mechanisms that vary from changes in hormonal content, production of volatile compounds, increasing nutrient availability, or enhancing abiotic stress tolerance (Ruzzi and Aroca, 2015). In addition, some native plant epiphytic microbiota can be used for bio-control of foodborne pathogens (Lopez-Velasco et al., 2012).

Plant growth-promoting (PGP) activity of epiphytic microbes can be affected by the environmental conditions, including the exposure to biostimulants/fertilizers or to their degradation products (Timmusk et al., 2017; Thapa et al., 2018). Protein hydrolysates (PHs) and other plant extracts are widely used as plant biostimulants for their positive effects on plant growth and nutrition (Colla et al., 2014). In the last years, the use of biostimulants to promote plant growth has been widely studied (Parrado et al., 2007; Kowalczyk et al., 2008; Ertani et al., 2009; Gurav and Jadhav, 2013; Colla et al., 2015). Foliar and root applications have been shown to enhance the uptake of both macro and micronutrients (Ertani et al., 2009, 2012; Colla et al., 2015; Halpern et al., 2015) and to increase plant tolerance to environmental stress conditions. Biostimulant action of vegetal-derived products has been ascribed to the supply of bioactive compounds like amino acids, peptides, carbohydrates, humic substances, lignosulphonates, and phytohormones (Du Jardin, 2015). These bioactive compounds can be available in the plant-derived biostimulant or can be generated after foliar spray or substrate drench through the microbial activity.

Despite the use of biostimulants has been increasing and has become a common practice in the sustainable agriculture, little information is available on the effect of these products on the epiphytic bacterial microbiota. PHs and vegetal extracts-based products contain a wide range of compounds, as simple or complex carbohydrates and soluble organic nitrogen (Trouvelot et al., 2014), that can be utilized by both plants and bacteria as a source of carbon, nitrogen, and energy (Farrell et al., 2013). Therefore, foliar application of biostimulants can affect the epiphytic microbiota enhancing the development of bacteria that can be beneficial, neutral, or detrimental to plants (Colla et al., 2017a). Structural and functional modifications in the plant-associated microbiota have a crucial impact on the ecosystem, altering antagonistic and synergistic interactions among microorganisms and improving the fitness of the host by enhancing plant metabolic capacity, uptake of nutrients, and response of the plant to abiotic and biotic stresses (Kumar and Verma, 2018; Valencia et al., 2018). The exposure of plants to harsh environmental conditions can strongly influence the structure and composition of its microbiota, but this effect can be counteracted by enhancing the growth and survival of stress-tolerant PGP bacteria (Gouda et al., 2018). Therefore, growth stimulation of beneficial epiphytic microbes might be one of the different modes of action of some plant biostimulants.

In the case of amino acids and small peptide-based products, several studies on plant growth have been reported (Mladenova et al., 1998; Qurartieri et al., 2002). Kauffman et al. (2005) showed a growth-regulator activity of Foliar, an array of free amino acids and other organic constituents, on turf. The assessment of effects of foliar-applied agricultural products on plant epiphytic microbes guarantees the consumers harmless final product, especially for leafy vegetables such as lettuce. In lettuce, the diversity and abundance of epiphytic microbial community depend on the cultivation environment, i.e., phyllosphere microbiota from laboratory-grown plants is distinct from that colonizing plants grown in the field (Williams and Marco, 2014), and can be affected by the climatic variations such as radiation, rainfall, wind, and temperature (Medina-Martínez et al., 2015). The alteration of the composition of epiphytic microbial community of lettuce may lead to the promotion of beneficial microbes which can compete for the nutrient sources with enteric pathogens (Cooley et al., 2006) and perhaps phytopathogens.

We hypothesized that foliar applications of commercial products containing vegetal-derived bioactive compounds can enhance crop growth not only directly through the activity of signaling molecule such as peptides (e.g., short peptides such as root-hair promoting peptides), amino acids (e.g., glutamate), and phytohormones (e.g., auxins, cytokinins) but also indirectly by changing the microbial community in the phyllosphere. Vegetal-derived organic compounds sprayed on the leaf surface may be used as carbon and energy sources by beneficial microorganisms to synthesize new organic compounds including plant growth stimulating molecules (e.g., hormones) and/or toxic substances for plant pathogens. Moreover, vegetal-derived biostimulants are often enriched with copper or other micronutrients to improve plant nutrition and crop protection against pathogens. Several authors demonstrated that copper could alter the structure and function of the soil microbial communities, resulting in the selection of copper tolerant and copper resistant strains (Berg et al., 2012; Griffiths and Philippot, 2013; Nunes et al., 2016). Similarly, the addition of mineral elements to vegetal-derived biostimulants may also affect the microbial community in the phyllosphere and then the biostimulant activity of the product. To our knowledge, no information is available in the scientific literature on the influence of foliar applications of vegetal-derived bioactive compounds alone or enriched with micronutrients on bacterial community in the phyllosphere. Starting from the above considerations, a lettuce trial was carried out under greenhouse conditions to evaluate the influence of foliar sprays with three different commercial products containing vegetal-derived biostimulant compounds [tropical plant extract enriched with micronutrients (PE) “Auxym”; vegetal-derived PH “Trainer”; vegetal derived PH enriched with copper “Scudo”] on bacterial community from lettuce leaves in order to determine whether the product-mediated growth stimulation of certain epiphytic microbes contributes to the plant growth promoting properties of these products.

## Materials and Methods

### Growing Conditions and Treatments

The trial was conducted, 2012, in a 300-m^2^ polyethylene greenhouse situated at the Experimental Farm of Tuscia University, central Italy (latitude 42° 25′ N, longitude 12° 08′, altitude 310 m). Plants were grown under natural light conditions. The mean values of day/night air temperature and relative humidity and their standard deviations were 15.6 ± 1.3/22.7 ± 4.5°C, and 55.1 ± 8.2/80.0 ± 8.9%, respectively. Lettuce seeds (*Lactuca sativa* L. cv “Green Salad Bowl,” SAIS S.p.A., Cesena, Italy) were sown on April 9 in polystyrene plug trays filled with vermiculite at a plant density of 720 plants m^-2^. The floating raft system consisted of the polystyrene plug trays floating in plastic tanks with a constant volume of 60 L of aerated nutrient solution. An air compressor maintained the dissolved oxygen content above 6 mg/L. The composition of the nutrient solution in all treatments was: 10 mM NO_3_-N, 1.5 mM P, 4.5 mM K, 10 mM Ca, 5.0 mM S, 2 mM Mg, 20 μM Fe, 9 μM Mn, 0.3 μM Cu, 1.6 μM Zn, 20 μM B, and 0.3 μM Mo. The electrical conductivity and pH of the nutrient solutions in all treatments were 2.0 ± 0.2 dS m^-1^ and 6.0 ± 0.3, respectively. To prevent large fluctuation in the nutrient concentrations, electrical conductivity, and pH, the nutrient solutions in all treatments were renewed from all tanks weekly.

After 14 days from emergence, plants were sprayed with 2.5 ml L^-1^ of a vegetal-derived PH (“Trainer”), or 1.0 ml L^-1^ of a copper-based fertilizer (“Scudo”), or 1 ml L^-1^ of a plant extract (“Auxym”).

The plant extract “Auxym” (PE) is a commercial vegetal-derived biostimulant produced through water extraction and fermentation of tropical plant biomass. It contains mainly phytohormones with an auxin:cytokinin ratio 6:1, amino acids, vitamins, and microelements (Table 1; Colla et al., 2017b).

**Table 1:** Main components of products tested in the lettuce trial.

	Tropical plant extract enriched	Vegetal-derived protein	Vegetal-derived protein hydrolysate
Class of compound	with micronutrients (PE)	hydrolysate (PH)	enriched with copper (Cu-PH)
Phytohormones (mg kg^-1^)	Auxins (1.81)	ND	ND
	Cytokinins (0.29)		
Organic nitrogen compound (g kg^-1^)	Amino acids and peptides (51.9)	Free amino acids and peptides (310)	Free amino acids and peptides (150)
Vitamins (g kg^-1^)	Niacin (3.3)	ND	ND
	Vitamin C (1.0)		
	Vitamin E (0.4)		
	Thiamine (0.3)		
	Pyridoxine (0.3)		
	Riboflavin (0.2)		
Micronutrients (g kg^-1^)	Fe-EDTA (6.0)	Traces	Cu-complexed with amino acids and peptides (90) and traces of other micronutrients
	Mn-EDTA (6.0)		
	Zn-EDTA (4.0)		
	Cu-EDTA (2.0)		
	B-H_3_BO_3_ (4.0)		


*ND = not detected.*

The legume-derived PH “Trainer” is a commercial biostimulant obtained through enzymatic hydrolysis of proteins derived from legume seeds. It contains mainly free amino acids, and soluble peptides with the following aminogram (g kg^-1^): Ala (12), Arg (18), Asp (34), Cys (3), Glu (54), Gly (12), His (8), Ile (13), Leu (22), Lys (18), Met (4), Phe (15), Pro (15), Thr (11), Trp (3), Tyr (11), Val (14). The total content of micronutrients being below 0.05 g kg^-1^ can be considered negligible (Table 1). It has been shown that small peptides and single amino acids present in this PH exhibit auxin-like and gibberellin-like activities (Colla et al., 2014).

“Scudo” is a copper-based fertilizer (Cu-PH) containing copper complexed with peptides, and amino acids (90 g kg^-1^ of copper). Scudo (Cu-PH) contains free amino acids, and soluble peptides with the same composition of Trainer (PH; Table 1). Trainer, Scudo, and Auxym were manufactured by Italpollina S.p.A., Rivoli Veronese, Italy.

Foliar applications were repeated three times, at weekly intervals and, in each application, control plants were sprayed with the same amount of water used for the three vegetal-derived products. The four treatments were arranged in a randomized block design with three replicates per treatment (total of 12 plots). The number of plants per experimental plot was 84. Each plot included a polystyrene plug trays floating in a plastic tank. One day and seven days after the last treatment, six leaves (two from three independent plants) were sampled in each plot providing a total of 18 leaves per treatment (=6 leaves per plot × 3 replicates) per time point. Before the end of the trial (May 14, 35 days after sowing), the Soil-Plant Analysis Development (SPAD) index was recorded on lettuce leaves. A portable chlorophyll meter (SPAD-502, Minolta corporation, Ltd., Osaka, Japan) was used to measure the relative leaf chlorophyll concentration as a rational unit. Measurements were made at the central point of the leaflet between the midrib and the leaf margin of the second leaf starting from the apical shoot. Twenty random readings per plot were taken for each replicate and averaged to a single SPAD value; therefore, there were a total of three averaged SPAD values for each treatment. At end of the trial (May 14, 35 days after sowing), 20 plants per plot (single replicate) were harvested and the mean fresh weight of shoot biomass was determined from the 60 plants harvested from each treatment.

### Isolation of Culturable Bacteria From Lettuce Phyllosphere

Epiphytic bacterial populations from treated and no-treated plants were recovered incubating six leaves per replicate in 20 mL of saline solution (0.9% w/v NaCl) for 60 min, under shaking condition (180 r/min). There were three replicates for each treatment with a total of 18 leaves per treatment per time point. Cell suspensions were serially diluted onto LB agar plates (Sambrook and Russel, 2001) for counting of predominant culturable bacteria and individual colonies were then picked and streaked on fresh LB plates for further characterization.

### DNA Extraction

At each time point, metagenomic DNA used for T-RFLP and NGS analysis was prepared from three biological replicates for each treatment and from each biological replicate, two replicate extractions were performed. DNA was extracted from cells collected by centrifugation (13,000 r/min for 10 min) from saline solution used for leaf washing (six leaves for each replicate, two leaves from three independent plants per plot). Total DNA of culturable isolates was obtained from cells grown overnight on LB medium. DNA was extracted using PureLink Genomic DNA Mini Kit (Thermo Fisher Scientific, Italy) following the manufacturer protocol for Gram-positive bacteria^1^. The quantity and quality of isolated DNA was measured using a Qubit dsDNA HS Assay kit (Thermo Fisher Scientific, Italy) and agarose gel electrophoresis, respectively.

### Molecular Identification of Culturable Bacteria

Two to four isolates for each morphotype were selected for the molecular analysis. The culturable isolates were characterized by amplifying, sequencing, and analyzing the 16S rRNA gene. Universal primer 63F (5′-CAGGCCTAACACATGCAAGTC-3′) and 1389R (5′-ACGGGCGGTGTGTACAAG-3′) were used to generate amplicons (about 1400 bp) that were cloned into the pGEM-Teasy vector (Promega, Madison, WI, United States) and sequenced using MACROGEN commercial service (Amsterdam, Holland).

All 16S rRNA sequences from isolates with the same morphotype were identical and only sequences that could be shown to be derived from independent templates were analyzed. The 16S rRNA sequences were compared with those of all known bacterial species available in the GeneBank database^2^ to identify potential phylogenetic relationships. All sequences were aligned using Clustal Omega (Sievers et al., 2011) and the unrooted phylogenetic tree was constructed using the neighbor-joining program contained in the PHYLIP phylogeny inference package (ver 3.6). The confidence values of the branches were determined by performing a bootstrap analysis based on 1000 replicates and the phylogenetic tree was displayed using iTOL (Letunic and Bork, 2006).

### T-RFLP Analysis

To ensure that the Terminal Restriction Fragment Length Polymorphism (T-RFLP) analysis was not biased by biological sample, DNA extraction, and amplicon library preparation, two DNA pools, each consisting of equimolar DNA samples from biological replicates of each treatment, were prepared. Thus, the profile of the epiphytic bacterial community was derived analyzing 18 leaves from nine plants for each treatment. PCR amplification targeting the 16S rRNA gene was carried out using primer 8F (5′-AGAGTTTGATCCTGGCTCAG-3′), fluorescently labeled at the 5′ end with 6-FAM (6-carboxyfluorescein), and 1387R (5′-GGGCGGWGTGTACAAGGC-3′), with three replicates per DNA pool. PCR replicates were purified, using the Promega Wizard (Promega, Madison, WI, United States), quantified, using Qubit dsDNA HS Assay kit (Thermo Fisher Scientific, Italy), and pooled into a single tube to represent each amplicon library in equimolar amounts. Fluorescently labeled products were digested with 10 U of restriction enzyme *Ssp*I or *Avr*II (Thermo Fisher Scientific, Italy) by following the manufacturer’s instructions. Digested products were purified and analyzed on an ABI3730 capillary sequencer in genotyping mode with the size standard ROX-labeled GS500. Only peaks that achieved a prevalence of more than 1% have been considered. Replicate T-RF profiles of each DNA pool and T-RF profiles of distinct DNA pools from samples of each treatment gave reproducible fingerprints. Consensus profiles were created as suggested by Dunbar et al. (2001), using the average values for peak heights. Total richness (S), Shannon’s diversity index (H), and Simpson’s evenness index (E) were calculated using PRIMER (v7, PRIMER-E Ltd., Plymouth, United Kingdom).

### NGS Analysis

The V4 hypervariable region of the 16S rRNA gene was amplified using modified universal bacterial primer pairs 515F/806R designed for use with the Illumina platform (forward primer: 5′-TCGTCGGCAGCGTCAGATGTGTATAAGAGACAGGTGCCAGCMGCCGCGGTAA-3′; reverse primer: 5′-GTCTCGTGGGCTCGGAGATGTGTATAAGAGACAGGGACTACHVGGGTWTCTAAT-3′), with three replicates per DNA pool. PCR replicates were purified, quantified, and pooled in equimolar amounts as described before. Subsequently, amplicons were indexed and sequenced according to the Illumina MiSeq 16S Metagenomic Sequencing Library Preparation protocol^3^. Sequencing was performed on the Illumina MiSeq platform (Illumina, San Diego, CA, United States) at Molecular Digital Diagnostics S.r.L. (Viterbo, Italy).

The Quantitative Insights Into Microbial Ecology (QIIME, v1.9.0; Caporaso et al., 2010) software was used to analyze the 16S rRNA sequence generated from paired-end amplicon sequencing. Paired-end reads were merged with PEAR (Zhang et al., 2014), setting a *p*-value cutoff of 0.05. Chimeras were detected and filtered from the paired-end reads using USEARCH (v6.1; Edgar, 2010). Operational taxonomic units (OTUs) were assigned to the reads using an open reference approach with UCLUST algorithm (Edgar, 2010) against the SILVA database release 132 that was clustered at 97% identity.

Based on the genus-level classification, principal component analysis (PCA) was performed to evaluate the similarity among various metagenomic communities. The PCA plots were displayed using PAST 3 (Hammer et al., 2001). The relative microbial abundance of all samples was summarized in a taxa plot.

### Indole Acetic Acid Production

Estimation of extracellular indole acetic acid (IAA) was determined using the colorimetric method described by Patten and Glick (2002). Bacteria were grown for 16 h at 30°C, in LB medium (50 mL) without or with L-tryptophan (0.1% wt/vol). After growth, spent medium was recovered by centrifugation at 8000 r/min for 10 min and directly used for IAA quantification using Salkowski’s reagent (0.5 M FeCl_3_ in 35% perchloric acid). The mixture was incubated at room temperature for 30 min and absorbance of the developed pink color was read at 530 nm. IAA concentration in the culture was determined by using a calibration curve of pure IAA (Sigma–Aldrich, Italy), as a standard. All these experiments were carried out in triplicate.

### Phosphate Solubilization

Phosphate solubilization activity was determined on NBRIP medium agar plates containing insoluble Pi. NBRIP medium contained (per liter of distilled water): glucose, 10 g; MgCl_2_^.^6H_2_O, 5 g MgSO_4_^.^7H_2_O, 0.25 g; KCl, 0.2 g; (NH_4_)_2_SO_4_, 0.1 g; Ca_3_(PO_4_)_2_, 5 g (Nautiyal, 1999). Plates were incubated for 2–5 days at 30°C. Colonies with clear halos were considered as phosphate solubilizing colonies. Solubilization index (SI) was calculated as: SI = (colony diameter + halo zone diameter)/colony diameter (Premono et al., 1996). All these experiments were carried out in triplicate.

### Antimicrobial Activity

Evaluation of antimicrobial activity was performed on the following strains: *Erwinia amylovora* Ea273 strain (ATCC 49946); *Trichoderma reseii* DIBAF-10 an environmental strain isolated from poplar chips, *Trichoderma viride* T-67 strain (ATCC 28020), *Phytophthora cinnamomi* isolate 1, *Fusarium culmorum* isolate 485, *F. culmorum* isolate J1, *Fusarium oxysporum* isolate 2, and *Fusarium graminearum* isolate 3, were kindly supplied by Prof. G. Chilosi (DIBAF, University of Tuscia).

Production of diffusible compounds with antifungal activity was tested on Potato Dextrose Agar (PDA) using a dual-culture *in vitro* assay. First, a 5-mm-diameter mycelium disk from a 5-day-old fungal culture was placed on the surface of the agar plate in the center of the petri dish. Then, a bacterial suspension from an overnight culture on LB medium was streaked, on three sides, at a distance of about 3 cm from the fungus plug. Zones of inhibition were measured after 5 days of incubation at 30°C according to the method of Geels and Schippers (1983). Bacterial strains that caused an inhibition zone of at least 2 mm were judged as positive. All these experiments were repeated independently at least twice.

Production of bioactive volatile organic compounds (VOCs) with antimicrobial activity was assessed by the double plate technique. This analysis was carried out on *Bacillus* strains F13 and F14 which exhibited the best inhibition against phytopathogenic *Fusarium* and *Phytophthora* in dual-culture assay. One hundred microliters of a bacterial suspension from an overnight culture on LB medium was spread on a LB-agar petri dish and a 5-mm disk of a 5-days-old pure culture of the fungus was placed at the center of another Petri dish containing PDA. Both half plates were placed face to face, preventing any physical contact between the fungal pathogen and the bacterium, and sealed to isolate the inside atmosphere and prevent loss of volatiles formed during the growth. Each pair of plates was incubated at 30°C for 48 h and the growth of the pathogen was measured and compared to a control prepared in the same manner but without bacteria.

For the determination of the inhibitory effect of the bacterial isolates on pathogenic fungi, the percentage of inhibition of radial growth (PIRG %) was calculated according to the following formula 100 × (R1 - R2)/R1, where R1 and R2 are the radial growth of the pathogen in the absence and in the presence of the antagonist, respectively.

Antibacterial activity was assessed on plates according to Homma et al. (1989). Bacterial isolates were grown as spots on LB agar plates for 18 h and exposed to chloroform vapors for 30 min. After aeration, plates were covered with a suspension of *E. amylovora* Ea273 strain, obtained by mixing 5 mL of diluted LB soft agar (0.6%) with 200 μL of a stationary phase culture. Plates were incubated at 30°C until inhibition halos were detected.

Fungal and bacterial inhibition assays were repeated independently at least twice.

### Nucleotide Sequence Accession Numbers

Sequences from independent templates/clones and amplicon libraries were deposited in GenBank under accession number: MH329697; MH338201; MH341118; MH375452; MH375453; MH375462; MH375472- MH375478; MH3755548; MH375601; MH375635; MH375635; MH375636; MH375453; MH376404; MH376429; MH376688; MH3756691; MH376690; MH376691; MH379797.

### Statistical Analysis

Analysis of variance of the data was calculated using the software package, SPSS 10 for Windows, 2001. Tukey HSD test was performed at *p* = 0.05 on each of the significant variables measured. PCA was carried out compare NGS data obtained with samples collected 1 day after the last treatment. The PCA outputs include variable loading to each selected component and treatment component scores.

## Results

### Effects on Plant Growth and Epiphytic Bacteria

Effects of vegetal-derived products on plant growth and composition of bacterial communities associated with lettuce leaves were determined on 5-weeks-old plants treated for 21 days with commercial extracts or hydrolysates of vegetal proteins. Leaf chlorophyll content expressed by the SPAD index was significantly increased by PE and PH (avg. 24.9) in comparison with Cu-PH and untreated control (18.9; Table 2). Fresh shoot biomass was also significantly enhanced by foliar applications of plant extracts or hydrolysates of vegetal proteins (avg. 6.69 g/plant) in comparison with untreated control (6.05 g/plant). After 1 day from the foliar application, the highest culturable aerobic epiphytic bacteria were observed in PE treatment whereas Cu-PH gave the lowest value. However, after 7 days from the foliar application the culturable aerobic epiphytic bacteria showed a different trend with the highest value in Cu-PH treatment (Table 2). Similarly, to fresh shoot biomass, leaf fresh weight sampled for the determination of abundance of culturable aerobic epiphytic bacteria was highest in lettuce plants treated with commercial products (Table 2).

**Table 2:** Crop parameters and cultivable epiphytic bacteria from lettuce plants treated and no treated with the three commercial products.

	Leaf SPAD	Shoot fresh	Leaf fresh	
Treatment	index	weight (g/plant)	weight (g/leaf)	Aerobic cultivable bacteria [CFU (^∗^10^3^)/g biomass]	
				
				day after treatment	7 days after treatment
No-treated	18.0 ± 2.4^b^	6.05 ± 0.28^b^	1.03 ± 0.01^d^	2.48 ± 0.03^b^	1.11 ± 0.01^b^
PE	24.6 ± 0.6^a^	6.62 ± 0.20^a^	1.23 ± 0.06^b^	4.92 ± 0.02^a^	0.92 ± 0.01^b^
Cu-PH	19.9 ± 2.9^b^	6.78 ± 0.14^a^	1.27 ± 0.04^a^	0.10 ± 0.05^c^	17.90 ± 0.80^a^
PH	25.3 ± 1.6^a^	6.68 ± 0.21^a^	1.19 ± 0.04^c^	1.76 ± 0.05^b^	0.98 ± 0.01^b^
Significance^a^	^∗^	^∗∗^	^∗∗^	^∗∗^	^∗∗^


*PE = tropical plant extract; PH = protein hydrolysate; Cu-PH = copper-based PH. Values ( ± SD) with no letter in common significantly differ at *p* ≤ 0.05 (Tukey HSD test).^a∗^,^∗∗^ Significant at *P* ≤ 0.05 and 0.01, respectively.*

Differences in the culturable count at 1 day were less pronounced when no-treated samples were compared to those collected from leaves of PE- or PH-treated lettuce. As shown in Table 2, the number of culturable epiphytic bacteria increased twofold in PE-treated plants (from 2.48 ± 0.03∗10^3^ to 4.92 ± 0.02∗10^3^ CFU/g of biomass) and had a slight decrease (about 0.3-fold) in samples from PH-treated plants (from 2.48 ± 0.03∗10^3^ to 1.76 ± 0.05∗10^3^ CFU/g of biomass).

In no-treated and in PE- or PH-treated plants, the abundance of culturable bacteria significantly decreased over the time and, 7 days after the last treatment, reached the same final value (between 0.92 ± 0.01^∗^10^3^ and 1.11 ± 0.01^∗^10^3^ CFU/g of biomass; Table 2).

### Molecular Characterization of Culturable Bacteria

Based on colony morphology, we identified 23 morphotypes which were differentially distributed in the epiphytic population collected from leaves of treated and no-treated lettuce (Table 3). Only seven morphotypes were present in the aerobic bacterial population from no-treated control plants, while the larger number of morphotypes occurred in PH-treated lettuce (15 out of 23; Table 3).

**Table 3:** Epiphytic bacteria isolated from no-treated and treated lettuce leaves and identified by 16S rRNA sequencing.

Taxonomic affiliation		Morphotype	Biostimulant				T-RF (bp)	
			
Family	Genus		No-treated	PE	PH	Cu-PH	*Ssp*I	*Avr*II
Enterobacteriaceae	*Pantoea*	C1	-	+	+	-	355	259
	*Enterobacter*	C4	-	+	+	-	355	259
		C6	-	-	+	-	355	259
Bacillaceae	*Exiguobacterium*	F11	+	+	+	-	No cut	
		F15	-	-	+	-	No cut	
		C2	+	+	+	-	No cut	
	*Bacillus*	C5a	-	-	+	-	174	No cut
		C5b	-	+	-	-	174	No cut
		F12	-	-	-	+	No cut	
		F13	-	+	+	+	No cut	
		F14	+	-	-	-	No cut	
Pseudomonadaceae	*Pseudomonas*	C3	+	+	+	+	351	107
		C7	+	+	+	+	351	107
		F1G	-	+	+	-	354	107
		F4	-	+	+	-	354	107
		F5	+	-	+	+	351	107
		F9	-	-	+	-	354	218
		F16	+	+	-	-	351	218
Moraxellaceae	*Acinetobacter*	F2	-	-	+	+	351	No cut
		F7	-	+	-	-	351	No cut
Sphingobacteriaceae	*Sphingobacterium*	F8	-	-	-	+	361	No cut
Micrococcaceae	*Micrococcus*	F3	-	-	-	+	351	No cut
		F10	-	-	-	+	351	No cut


*(+) = present; (-) = absent/no-detectable.*

**FIGURE 1:**
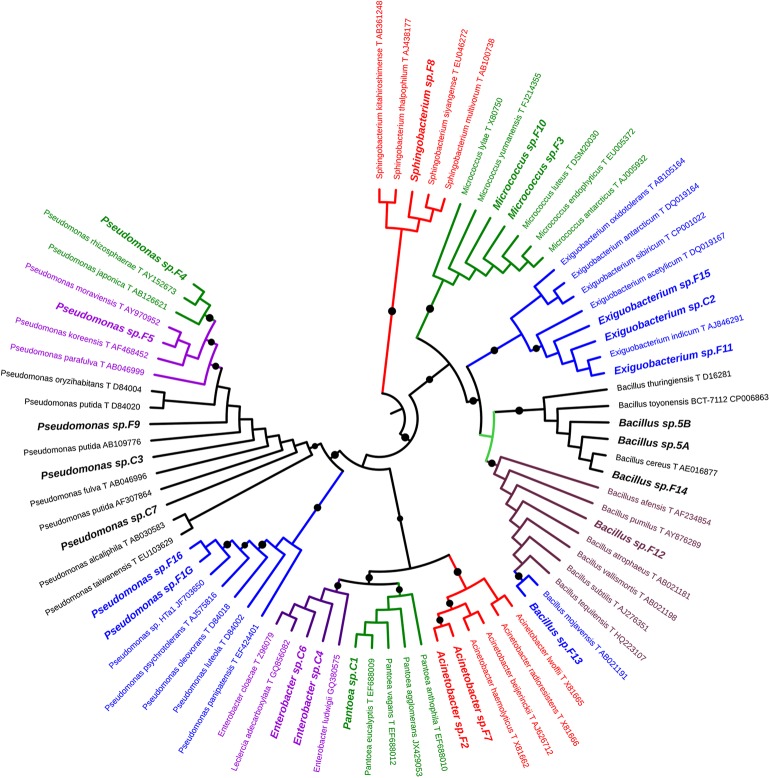
Phylogenetic tree of 16S rRNA gene sequences showing the relationship among the isolated from lettuce samples and the related genera.

For each morphotype, at least two independent colonies were characterized to the genus/species level using 16S rRNA gene as DNA barcode (see section “Materials and Methods”). Sequence data of 16S fragments were used to generate a phylogenetic tree to evaluate the genetic relatedness among these bacteria and known species (Figure 1). All strains exhibiting the same morphotype had identical 16S rRNA gene sequences (not shown). The 23 morphotypes belonged to six family and eight different genera of which *Pseudomonas*, *Bacillus*, and *Exiguobacterium* were the most recurrent. As shown in Figure 1, all *Exiguibacterium* strains clustered with *Exiguibacterium indicum*; *Bacillus* strains clustered into different clades corresponding to *Bacillus cereus* group (strain 5a, 5b, and F14), *Bacillus pumilus* (strain F12), and *Bacillus mojavensis* (strain F13); *Pseudomonas* strains could be organized in four different groups clustering with *Pseudomonas putida* (strain C3, C7, and F9), *Pseudomonas psychrotolerans* (strain F1G and F16), *Pseudomonas rhizosphaerae* (strain F4), and *Pseudomonas moraviensis* (strain F5), respectively (Figure 1).

### Molecular Characterization of Bacterial Epiphytic Community

The structure of the total epiphytic bacterial community was evaluated using a molecular approach based on the T-RFLP of 16S rRNA gene (Osborn et al., 2000). To identify T-RFs in the community profiles, *Avr*II and *Ssp*I fingerprints of target gene from culturable bacteria reported in Table 3 were generated. Completed digestion of PCR products gave T-RF of: 107 (218) bp for *Avr*II and 351 (354) bp for *Ssp*I on *Pseudomonas* DNA; 351 bp (*Ssp*I) on *Acinetobacter* and *Micrococcus* DNA; 259 (*Avr*II) and 355 bp (*Ssp*I) on *Enterobacter*/*Pantoea* DNA; 361 bp (*Ssp*I) on *Sphingobacterium* 16S gene. PCR product from *Bacillus* C5a and C5b generated a single peak of 174 bp after *Ssp*I digestion. Both enzymes do not generate detectable T-RFs using, as a template, DNA from *Exiguobacterium* and other *Bacillus* isolates.

**FIGURE 2:**
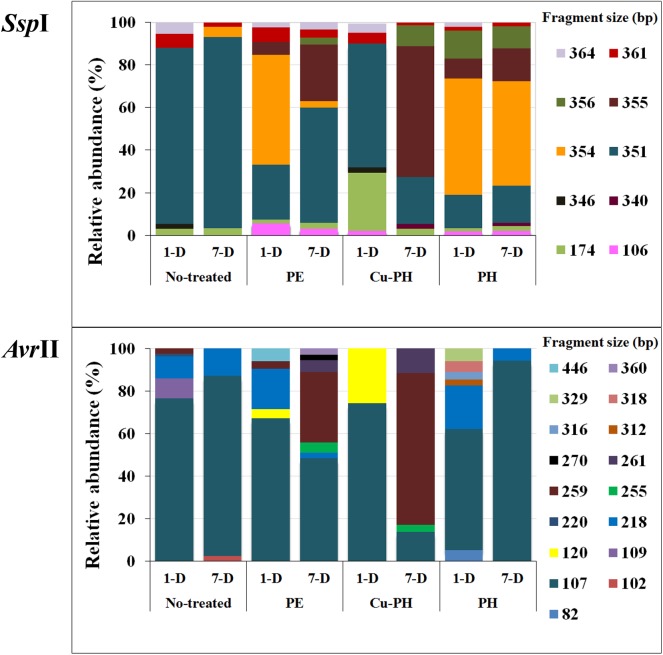
Percentage of relative abundance of T-RFs after *Ssp*I and *Avr*II digestion among no-treated and PE-, Cu-PH-, and PH-treated samples at 1 and 7 days after the last treatment. The numbers indicate the size (in bp) of the T-RF fragments.

As shown in Figure 2, *Ssp*I and *Avr*II digestions of amplicons from epiphytic bacterial community gave a total of 10 and 17 peaks, respectively. Differences in the absolute number and in the relative height of discernible peaks could be seen comparing no-treated and treated samples, as well as samples collected 1 or 7 days after the last treatment. Differences in T-RF profiles of samples collected at 1 and 7 days were observed with both treated and no-treated plants. Three *Ssp*I T-RFs (351, 361, 364 bp) and one *Avr*II T-RF (107 bp) occurred in all samples, albeit the relative abundance (RA) of each of these phylotypes was significantly different in the single T-RFs pattern. In T-RF profiles of no-treated samples (1 and 7 days), a lower number of peaks were observed and with both enzymes 80% of the total peak area was associated to a single peak (351 bp in *Ssp*I profiles, 107 bp in *Avr*II profiles). In T-RF profiles of treated samples (1 and 7 days), specific additional peaks were observed at: 106, 355, and 356 bp for *Ssp*I, 120, 255, 259, and 261 bp for *Avr*II.

**FIGURE 3:**
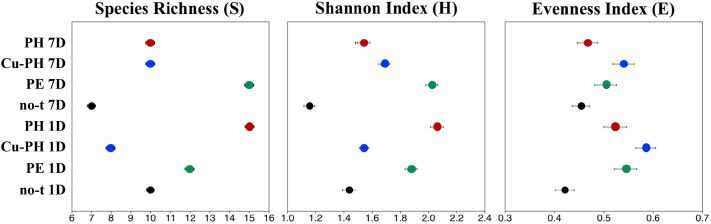
Values for ecological diversity indices of total richness (S), Shannon–Weaver diversity (H), and evenness (E) obtained by using combined *Ssp*I and *Avr*II T-RFLP data of samples collected 1 or 7 days after the last treatment.

To describe the changes in the dominance among the phylotypes, the ecological diversity indices were calculated combining the data of both enzymes (Figure 3). The maximal number of species determined by T-RFLP analysis (15) was found in 1-day PH-treated samples and 7 days after the last treatment in samples from PE-treated plants (Figure 3). As shown in Figure 3, for PE and Cu-PH we observed an increase in species richness from 1 to 7 days. In contrast, in no-treated and PH-treated samples, we observed an overall reduction in the species richness over the time. In samples collected 1 and 7 days after the last treatment from PE- and PH-treated plants, the diversity (H) and evenness (E) were much higher than in no-treated plants (Figure 3). Interestingly, in samples collected 1 day after the last treatment with Cu-PH, we observed a reduction in the species richness (S), as well as an increase in the evenness compared to no-treated plants (Figure 3). These observations indicated that, on leaves of Cu-PH-treated plants, a lower number of species were present but a more equitable distribution in species abundance occurred.

**FIGURE 4:**
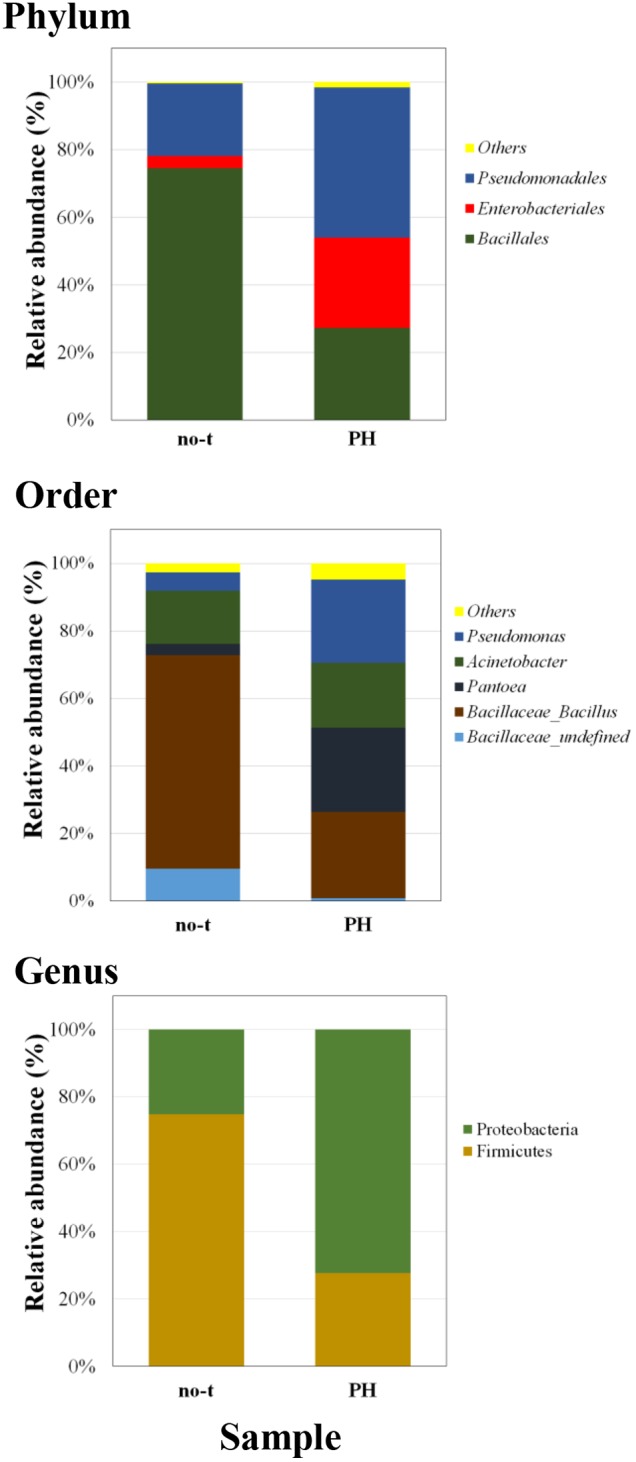
Barplots illustrating the diversity and relative abundances at the phylum, order, and genus levels in PH-treated and no-treated samples collected 1 day after the last treatment. The taxonomy is constructed with the database Silva with a confidence threshold of 97%.

To gain more insights about the effect of vegetal PHs on the structure of bacterial community of lettuce phyllosphere, samples collected one day after the last treatment were also analyzed using a next generation sequencing (NGS) approach. This analysis was only carried out on PH-treated and no-treated plants for two major reasons: the treatment with PH gave the highest species diversity (Figure 3); PH does not contain inorganic compounds, such as copper salts (Cu-PH) and micronutrients (PE), whose combined effect with vegetal PHs could not be easily uncoupled. NGS analysis based on the sequencing of V3–V4 region of 16S rDNA gene allowed us to generate a number of reads per sample comprised between 120,000 and 150,000 (not shown). Approximatively 90% of raw reads per sample passed merging, trimming, and chimera filtering steps and were analyzed for OTU search. In both samples, two phyla accounted for 90% of total sequence reads with the majority belonging to *Firmicutes* (74.7% of total bacteria in no-treated samples) or *Proteobacteria* (72.2% in samples from PH-treated plants: Figure 4). In PH-treated (compared to no-treated) samples, we observed a reduction of *Bacillales* (from 74.5 to 27.3% of total bacteria) and an increase in *Pseudomonadales* (from 21.5 to 44.4%) and *Enterobacteriales* (from 3.6 to 26.7%; Figure 4). Other orders that occurred as minor forms in PH-treated (and were not detectable in no-treated) samples comprised *Sphingomonadales*, *Flavobacteriales*, *Micrococcales*, and *Pasteurellales*.

A deeper phylogenetic classification of the reads at genus level (Figure 4) revealed that the most over-represented genera in PH-treated samples were *Bacillus* (25.5% of total bacteria), *Pantoea* (24.9%), *Pseudomonas* (24.6%), and *Acinetobacter* (19.3%). In no-treated samples, *Bacillus* and related genera represented 73.1% of total bacteria, major genera under the order of *Pseudomonadales* were *Acinetobacter* (15.8% of total bacteria) and *Pseudomonas* (5.4%), whereas *Enterobacteriales* included bacteria belonging to *Pantoea* genus (3.3%; Figure 4).

**FIGURE 5:**
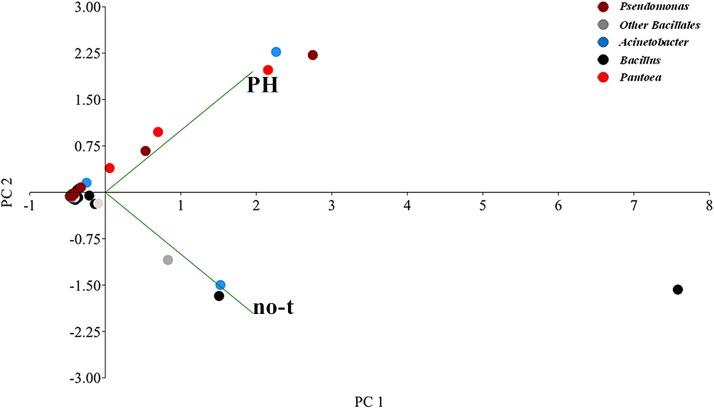
Genus-level principal component analysis (PCA) comparison of the normalized bacterial OTU data. The PCA axes differentiate the PH-treated and no-treated samples according to their microbial composition. Different genera are denoted with different colors. Principal Component 1 and 2 explained 78.4 and 21.5% of the total variations, respectively.

Principal component analysis analysis suggested substantial differences in the epiphytic microbial community between PH-treated and no-treated plants, as shown from the distribution in different zones of the PCA graph of data sets obtained from different samples (Figure 5). The two axes were involved in 99.9% of the total variance and could explain most variations in bacterial community structure. The first principal component (PC1) explained 78.4% and the second (PC2) 21.5% of the variance at genus level. Along the first axis, variability was mainly explained by an increase in the population of bacteria belonging to *Pantoea* and *Pseudomonas* genus (Figure 5). Variability along the second PCA axis corresponded to a decrease in the RA of some bacteria belonging to *Bacillus* and *Acinetobacter* genus (Figure 5).

### Phenotypic Characterization of Culturable Bacteria

Epiphytic isolates from lettuce plants were tested for features known to contribute to plant growth promotion, such IAA production and mineral phosphate solubilization, or plant growth protection against phytopathogens. Results reported in Table 4 indicated that about 30% of all isolates (7 out of 23) were able to solubilize mineral phosphate, with a SI ranging between 2.5 and 4.8.

**Table 4:** Plant growth-promoting (PGP) activity and inhibitory effect on the growth of plant pathogens of culturable epiphytic bacteria isolated from treated and no-treated lettuce plants.

Taxonomic affiliation	Morphotype	PGP activity		Biocontrol activity (% of inhibition)		
			
		SI^∗^	IAA^§^ (mg/L)	*F. oxysporum*	*P. cinnamomi*	*E. amylovora*
*Pantoea*	C1	0	106 ± 0.1	30–60%	<30%	>60%
*Enterobacter*	C4	0	0	0	0	0
	C6	0	0	0	0	0
*Pseudomonas*	F1G	4.3 ± 0.1	20 ± 0.1	30–60%	30–60%	0
	F4	0	0	0	0	0
	F5	4.8 ± 0.5	5.4 ± 0	<30%	<30%	0
	F9	0	0	<30%	<30%	0
	F16	3.0 ± 0.2	2.7 ± 0.1	30–60%	30–60%	30–60%
	C3	0	0	0	0	0
	C7	0	0	0	0	0
*Acinetobacter*	F2	2.5 ± 0.1	<5	0	0	0
	F7	0	<5	0	0	0
*Sphingobacterium*	F8	2.5 ± 0.1	0	0	0	0
*Bacillus*	C5a	0	0	>60%	<30%	>60%
	C5b	0	0	>60%	<30%	>60%
	F13	0	0	>60%	>60%	>60%
	F14	0	0	>60%	>60%	>60%
	F12	0	0	0	0	0
*Exiguobacterium*	F15	0	0	0	0	0
	F11	0	0	0	0	0
	C2	0	0	0	0	0
*Micrococcus* (2)	F3	2.7 ± 0.1	36 ± 0	<30%	<30%	>60%
	F10	4.7 ± 0	31 ± 0	0	0	0


*^∗^Phosphate solubilization index; ^§^ indole acetic acid production.*

About 35% of isolates (8 out of 23) produced indoleacetic acid in tryptophan supplemented LB medium. The highest level of IAA was obtained with *Pantoea* strain C1 (>100 mg L^-1^), and good levels of production were obtained with either *Pseudomonas* and *Micrococcus* (5–36 mg L^-1^) or *Acinetobacter* strains (≤5 mg L^-1^).

The antagonistic activity against phytopathogens was assayed using a dual culture technique (Oldenburg et al., 1996). Results reported in the Table 4 indicated that most *Bacillus* strains had a high antagonist activity against *F. oxysporum* and *E. amylovora*. *Bacillus* strains F13 and F14 also exhibited strong inhibitory activity against *P. cinnamomi* (Table 4). *E. amylovora* was also strongly inhibited by *Pantoea* sp. C1 and *Micrococcus* sp. F3 strains. Biocontrol activity against fungi belonging to *Fusarium* and *Phytophthora* genus although at lower inhibitory levels were also observed with *Pseudomonas* strains F1G and F16. The latter one also exhibited antagonistic activity against *E. amylovora* (Table 4).

### Antifungal Activity *in vitro*

The production of volatile metabolites active against fungi belonging to different *Fusarium* (*F. graminearum*, *F. culmorum*, and *F. oxysporum*) and *Trichoderma* (*T. viride* and *Trichoderma reesei*) species, was further investigated using *Bacillus* strains F13 and F14 which exhibited the best inhibition against phytopathogenic fungi in dual-culture assay.

**FIGURE 6:**
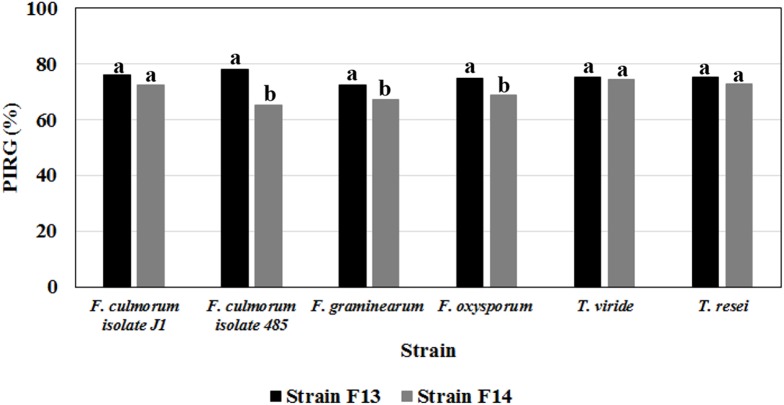
Antifungal activities of volatile metabolites produced on LB medium by *Bacillus* strain F13 and F14 against mycelial cultures of different phytopathogenic *Fusarium* and *Trichoderma* species. Data are expressed in percentage of inhibition of radial growth (PIRG); values with no letter in common significantly differ at *p* ≤ 0.05 (Tukey HSD test).

Results from double plate assays indicated that both strains produce volatile compounds with strong inhibitory activity (higher than 65%) against these phytopathogens (Figure 6). With F13 strain, PIRG % against *Fusarium* strains varied between 72.2 ± 0.3 (vs *F. graminearum*) and 78.0 ± 0.9 (vs *F. culmorum* isolate J1), whereas no significant difference was observed between PIRG values against *T. viride* and *T. reesei* (about 75%; Figure 6).

Interestingly, the metabolites produced by *Bacillus* F13 strain were stable and remained biological active over a wide range of pH (between 2 and 10) and temperature (between 4 and 100°C), showing no loss of activity even after autoclaving (data not shown).

## Discussion

The primary object of this work was to evaluate the effect of foliar applications of commercial products containing vegetal-derived bioactive compounds on the structure of epiphytic bacterial community and unforeseen implications on useful/deleterious bacteria enrichment.

Foliar applications of the three commercial products increased fresh weights of shoots and leaves in lettuce plants compared to control treatment; moreover, leaf chlorophyll content expressed by the SPAD index was enhanced by foliar applications of PE and PH in comparison with Cu-PH and control treatments. These results agree with the previous findings on the effect of PH and PE on corn, tomato and spinach (Colla et al., 2014, 2017b; Rouphael et al., 2018). Bioactive compounds such as amino acids, peptides, and phytohormones supplied by the commercial products may be responsible for the increase of foliar biomass (Table 2). To better understand the effect of plant-derived bioactive products on leaf-associated bacteria, data obtained using different approaches were combined to have insight into the distribution of specific taxa and on the potential ability of strains belonging to these taxa to promote plant growth and/or suppress diseases. PE was the unique tested product that determined a significant twofold increase in the number of cultivable epiphytic bacteria compared to samples from no-treated plants (Table 2). This positive effect of PE on plant growth could be due to the stimulation of cell proliferation induced by signaling compounds (e.g., phytohormones, amino acids), the better protection of cells from oxidative damage resulting from the supply of antioxidant compounds (vitamins), the enhancement of plant metabolism arising from the supply of micronutrients (Table 1; Colla et al., 2017b) as well as of its microbiota. Similar evidences were obtained analyzing differences in the bacterial communities between treated and no-treated plants 1 day after the last treatment. Ecological diversity measures indicated a significant increase (*P* < 0.05) in species richness, evenness, and diversity indices in PE-treated (as well as in PH-treated) compared to no-treated plants (Figure 3). This microbial diversity persisted for at least a week (Figure 3), whereas no significant difference in the abundance of culturable bacteria was observed comparing treated (PE or PH) and no-treated plants 7 days after the last treatment (Table 2). In contrast, the use of copper-containing products, such as Cu-PH, had a transient negative effect on the epiphytic bacteria, as demonstrated by the reduction, at 1 day, of total aerobic count (25-fold compared to no-treated plants Table 2) and total species richness (1.25-fold vs no-treated control; Figure 3). A similar effect on the reduction of the bacterial biodiversity was also observed by Almeida et al. (2017) studying the dynamic of the bacterial community in sediments exposed to copper and the use of these bacterial communities resistant to copper to improve phytoremediation of copper-contaminated sediments. A comparison of T-RFLP profiles (Figure 2) and indices of diversity and evenness (Figure 3) of samples from no-treated and Cu-PH-treated lettuce also demonstrated that Cu-PH strongly affected the structure of the bacterial epiphytic community. In fact, in samples collected 1 day after the last treatment, we observed a significant increase (*P* < 0.05) in both Shannon–Weaver diversity and evenness when plants were treated with Cu-PH (Figure 3). In particular, the RA (% of total peak height) of the 351-bp peak in *Ssp*I T-RFLP profile decreased from 83 (no-treated) to 58% (Cu-PH), while RA of *Ssp*I peak sized 174 bp, the representative peak of *Bacillus* morphotypes C5a and C5b (Table 3), increased ninefold (from 3 to about 27%; Figure 2). A similar stimulatory effect was also observed with other minor forms that were undetectable in the *Ssp*I T-RFLP profile of no-treated samples, such as 106-, 355-, and 356-bp peak. The latter *Ssp*I peaks were also detected in T-RFLP profiles of samples collected from PE- and PH-treated plants, indicating that plant-derived bioactive compounds have the ability to stimulate the growth of specific groups of bacteria related to morphotypes C1, C4, and C6 (*Ssp*I peak of 355 bp) belonging to *Enterobacte*r/*Pantoea* group (Figure 1) and to other not yet defined species (106- and 356-bp peaks). Interestingly, *Ssp*I peak sized 355 bp and the corresponding 259-bp *Avr*II peak became the most abundant ones (RA of 61 and 71%, respectively) in T-RFLP profiles obtained with samples collected from lettuce leaves 7 days after the last treatment with Cu-PH (Figure 2). Therefore, we can conclude that the use of vegetal-PHs and plant extracts can determine the enrichment of epiphytic bacteria related to *Enterobacter/Pantoea* group (Figure 1), and that this effect can be enhanced combining these products with copper. Interestingly, *Enterobacter/Pantoea* group includes beneficial bacteria with PGP traits and biological control against phytopathogens (Taghavi et al., 2009; Madhaiyan et al., 2010; Dutkiewicz et al., 2016; Singh et al., 2018), as well as strains having the ability to interfere with the quorum-sensing which control biofilm and EPS formation in some food-borne pathogens, such as *Yersinia enterocolitica* (Gopu et al., 2016). The ability of copper-tolerant bacteria to promote plant growth was also observed by Liu et al. (2014) analyzing bacteria isolated from mine tailings. Our observation is also in agreement with current literature on phytoremediation indicating that bacteria with PGP traits can facilitate the removal of inorganic contaminants stimulating either plant growth or phytoremediation activity (Glick, 2010; Glick and Stearns, 2011; Ullah et al., 2015).

As shown in Figure 3, effects on the structure of the epiphytic bacterial community were also observed treating lettuce with PE and PH. These products selectively stimulated the growth of *Pseudomonas*-related bacteria that were not detected in no-treated samples at 1 day (*Ssp*I T-RF sized 354 bp; Figure 2). This conclusion was supported by the information that in T-RFLP profiles peaks corresponding to *Pseudomonas* morphotypes F1G and F4 (*Ssp*I T-RF of 354 bp and *Avr*II T-RF of 107 bp; Table 2) and morphotype F9 (*Ssp*I T-RF of 354 bp and *Avr*II T-RF of 218 bp; Table 2) were predominant (>50%) in PE- (1 day) and PH-treated (1 and 7 days) samples (Figure 2). Interestingly, strains F1G, F4, and F9 are related to members of different *P. putida* subclusters (Figure 1) that have been characterized for their ability to solubilize inorganic phosphate, such as *P. rhizosphaerae* (Kwak et al., 2015), enhance plant growth or antagonize fungal phytopathogens, such as *Pseudomonas fulva*/*Pseudomonas parafulva*/*P. putida* (Pena et al., 2016), fix nitrogen and enhance nutrient uptake, such as *P. psychrotolerans* (Liu et al., 2017).

Peaks corresponding to *Pseudomonas* morphotypes C3, C7, and F5 (*Ssp*I T-RF of 351 bp and *Avr*II T-RF of 107 bp) and morphotype F16 (*Ssp*I T-RF of 351 bp and *Avr*II T-RF of 218 bp; Table 2) were predominant in no-treated and Cu-PH-treated at 1 day (RA of 83 and 58%, respectively), remained the major peaks in no-treated samples at 7 days (RA of 90%) and became the most abundant ones in PE-treated samples at 7 days (RA of 54%; Figure 2). These peaks were also present in T-RFLP profiles of samples from PH-treated lettuce, whereas they were not the most predominant ones and their RA (16–18%) did not change over the time (Figure 2). In conclusion, treatments with hydrolysates of plant proteins lead to changing patterns of *Pseudomonas* populations which are quite complex to analyze and might lead to a specific enrichment of strains, such as morphotype F1G, showing plant promoting traits (Table 4).

As mentioned before, peaks corresponding to *Enterobacter*/*Pantoea* morphotypes (*Ssp*I T-RF of 355 bp and *Avr*II T-RF of 259 bp; Table 2) were also present in PE- and PH-treated samples and their RA increased, over the time (1→7 day after the last treatment), from 6 to 27% in samples from PE-treated plants and from 9 to 16% in those from PH-treated plants (Figure 2).

Comparing differences in the relative fluorescence of *Avr*II peaks specific for *Pseudomonas* (107 and 218 bp) and 351-bp *Ssp*I peak belonging to *Pseudomonas* and other species, and considering that vegetal-derived PHs and plant extracts analyzed in this work also stimulated the growth of cultivable *Acinetobacter* and *Micrococcus* strains with a *Ssp*I T-RF peak of 351 bp (Table 3), we can postulate that, in samples from treated plants, the relative high values of fluorescence associated with the 351-bp *Ssp*I peak (compared to the combined fluorescens of 107- and 218-bp peak in *Av*rII profile) reflects the presence in the epiphytic community, in addition to *Pseudomonas*, of a more abundant population of bacteria belonging to *Acinetobacter* and *Micrococcus* genus. It is worth pointing out that *Acinetobacter* and *Micrococcus* strains isolated from treated plants (Table 3) have PGP traits, such as ability to produce plant-related hormones (IAA) or solubilize inorganic phosphate (Table 4) and, therefore, their enrichment can be valuable for the plant.

Strains with PGP traits were enriched with all commercial products examined in this work, albeit each product stimulated the growth of a specific group of microorganisms. For example, *Pseudomonas* and *Pantoea* strains able to produce high levels of IAA were specifically stimulated by PH or PE (morphotypes C1, F1G, and F5), whereas *Micrococcus* sp. strains (F3 and F10) able to produce IAA and solubilize inorganic phosphate were specifically enriched on plants treated with Cu-PH (Tables 3, 4). Other strains able to solubilize inorganic phosphate such as *Pseudomonas* sp. F1G or F5 were enriched by treatment with PE/PH or PH/Cu-PH, respectively.

The ability of vegetal-derived bioactive compounds to promote shifts in the composition of epiphytic bacterial communities, stimulating the growth of specific strains and increasing bacterial diversity, was confirmed by comparison of NGS data from no-treated and PH-treated plants (Figure 4). In fact, in samples collected 1 day after the last treatment, we observed that PH treatment altered the structure of the leaf-associated microbiome from phylum to genus level determining a reduction in the RA of bacteria belonging to *Bacillales* and, at the same time, an increase in the population of *Pseudomonas*, *Pantoea*, and other minor forms (Figure 4). Interestingly, the analysis at the level of individual OTUs demonstrated that PH specifically stimulates the growth of specific members of the epiphytic microbiota (Figure 5). This effect explains the increase in bacteria biodiversity, the alteration of the population of specific taxa, such as *Bacillus*, and the enrichment of rare species and specific strains which can play an important role in plant growth and protection (Shade et al., 2014; Jousset et al., 2017).

In agreement with data reviewed by Shafi et al. (2017), we demonstrated that microorganism belonging to *Bacillus* genus play an important role in lettuce microbiota as biocontrol agents (BCAs) against fungal and bacterial pathogens. Interestingly, the biocontrol activity is associated with strains related to *B. mojavensis* (F13) and *B. cereus* group (C5a, C5b, and F14; Figure 1), whose growth was stimulated by vegetal PHs and plant extracts (Table 3).

Among *Bacillus* strains isolated from treated plants, strain F13 exhibited the highest inhibitory activity (>71%) on the radial growth of all fungal pathogens tested in this work (Figure 6). The latter result is in agreement with data demonstrating that endophytic and epiphytic *B. mojavensis* strains have broad-spectrum antibacterial properties related to their ability to produce lipopeptides, surfactin and fengycin (Vágvölgyi et al., 2013; Kalai-Grami et al., 2014; Kim et al., 2015; Blacutt et al., 2016; Jasim et al., 2016). This wide range of metabolites can reduce pathogen attack by suppressing fungal growth or inducing the plant immune system (Khan et al., 2017). The ability of these bacteria to produce spore facilitates the use of *Bacillus* as bioinoculant.

## Conclusion

The present study revealed that vegetal PHs and extracts containing vegetal-derived bioactive compounds can stimulate the growth of epiphytic bacteria with PGP and/or biological control activity against pathogens. Metagenomic analysis also demonstrated that these products can stimulate the growth of rare members of the microbial community that can promote the biostimulant effects of vegetal PHs and have direct and indirect effects on the ecosystem functioning and the plant health.

This is the first report indicating that the use of this class of biostimulants can enrich autochthonous bacterial strains able to enhance plant growth.

## Author Contributions

MR, GC, and FL conceived the study and wrote the manuscript. AF and EBŠ contributed to corrections and suggestions. GC and EBŠ performed experiments on plants. FL isolated the culturable bacteria and extracted the metagenomic DNA. AF, MR, and FL performed the analysis of T-RFLP and NGS data and performed the phenotypic and molecular characterization of culturable bacteria. All authors read and approved the final manuscript.

## Conflict of Interest Statement

The authors declare that the research was conducted in the absence of any commercial or financial relationships that could be construed as a potential conflict of interest.

## Footnotes

1 https://assets.thermofisher.com/TFS-Assets/LSG/manuals/purelink_genomic_man.pdf

2 https://www.ncbi.nlm.nih.gov/genbank/

3 https://support.illumina.com/content/dam/illumina-marketing/documents/products/other/16s-metagenomics-faq-1270-2014-003.pdf
